# Draft genome sequence of *Bradyrhizobium* sp. 33ap4 associated with effective root nodulation in pigeon pea (*Cajanus cajan*)

**DOI:** 10.1128/mra.00573-25

**Published:** 2025-10-28

**Authors:** Francina Lebogang Bopape, Ahmed Idris Hassen, Eastonce Tendayi Gwata

**Affiliations:** 1 Agricultural Research Council, Plant Health and Protection (ARC-PHP) 527562 , Pretoria, South Africa; 2 Department of Plant and Soil Sciences, Faculty of Science, Engineering and Agriculture, University of Venda 56868, Thohoyandou, South Africa; University of Strathclyde, Glasgow, United Kingdom

**Keywords:** *Bradyrhizobium*, genome, chromosome, genes

## Abstract

*Bradyrhizobium* sp. 33ap4 is a free-living soil bacterium that was isolated from nodules on pigeon pea roots. This draft genome sequence revealed that the *Bradyrhizobium* strain consists of a 9.37-Mbp single chromosome with predicted nodulation and nitrogen fixation genes, DNA metabolism, and other stress response features.

## ANNOUNCEMENT

**Fig 1:**
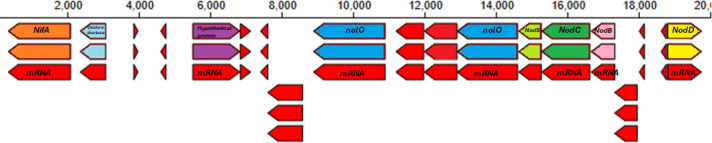
The chromosomal regions of annotated predicted genes for nodulation (*nodBCD*), nitrogen fixation (*NifA*), and the Nod factor regulating genes (*nolO*) of *Bradyrhizobium* sp. 33ap4 located on contig 28 and generated on the KBase platform.

*Bradyrhizobium* bacteria establish effective symbiotic associations with grain legumes by forming root nodules, thus providing fixed nitrogen and an alternative source of nitrogen to chemical fertilizers for farmers (1, 2). Here, we report the draft genome of a free-living *Bradyrhizobium* sp. strain 33ap4 that existed in virgin soil originating from South Africa (Fig. 1). The study also investigated the genes likely responsible for nodulation and nitrogen fixation. The soil, sampled from Thohoyandou Town (S22°57 E30°29), was used in a soil trap pot trial with the host plant pigeon pea. Nodules from pigeon pea roots were surface sterilized with 3.5% bleach and rinsed with sterile water five times. Nodules were squeezed with sterilized forceps and cultured on Yeast Mannitol Congo Red (YM-CR) agar that consists of mannitol 10 g, K_2_HPO_4_ 0.5 g, MgSO_4_.7H_2_O 0.2 g, NaCl 0.1 g, yeast extract 0.5 g, agar 15 g, Congo red 10 mL, and distilled water 1 L (3). The plates were incubated for 5–9 days at 28°C. Single colonies were selected and purified by re-streaking on YM-CR and incubated with the same conditions as described above. For DNA extraction, the cultures were transferred to Tryptone Yeast broth and incubated for 3 days on a shaker (150 rpm) at 28°C. The broth culture (1.5 mL) was used to extract DNA with the Wizard Genomic DNA Extraction Kit (Promega, Madison, USA). For the taxonomic identification using the 16S ribosomal RNA sequencing, we used the 16S primers: 27F (5′ AGA GTT TGA TCC TGG CTC AG 3′) and 1485R (5′ TAC CTT GTT ACG ACT TCA CCC CA 3′) (4). The rhizobium strain 33ap4, isolated from nodules on pigeon pea roots, showed a 99.76% 16S rRNA sequence similarity to *Bradyrhizobium* sp. with the accession number: MF140379.1. The BLASTn similarity search tool used on the NCBI Standard database nr/Core nucleotide database was accessed on 23 May 2025. For the draft genome sequencing, the extracted DNA was fragmented using a Covaris E220 Focused-Ultrasonicator (Thermo Fisher Scientific), followed by DNA library preparation using the MGIEasy Universal DNA Library Prep Kit (MGI Tech Co), and sequencing with the MGI DNBSEQ G400 platform using a PE150 sequencing cartridge at the Biotechnology Platform, ARC-Onderstepoort, South Africa. The number of read pairs obtained is 2,206,680 with a 200-bp read length. A total of 4.4 M spots were obtained, after which adapter sequences and low-quality bases were trimmed with Trimmomatic v.0.36 (5). The resulting raw reads were used for a *de novo* assembly with SPAdes v.3.15.3 (4) on KBase (6), which resulted in a draft genome with a total size of 9.4 Mb with 309 contigs, N50 of 109.4 kb, and L50 of 28 with a GC content of 63.5%. Scaffold was created with genome assembly software SSPACE v3.15.3 (7). The gene annotations were performed with the NCBI Prokaryotic Genome Annotation Pipeline (PGAP v6.9) (8, 9) that resulted in a total of 8,475 protein coding sequences, and 54 RNAs dispersed throughout the genome. The genome coverage was 100×, with a final scaffold number of 304, a total of 8,830 genes, and an assembly completeness of 98.57% (CheckM analysis v1.2.3) (10).

## Data Availability

This Whole Genome Shotgun project has been deposited at DDBJ/ENA/GenBank under the accession number JAUMTJ000000000, and the version described in this paper is JAUMTJ000000000.1. The SRA accession number for the raw data is SRX21096604, whereas the accession for the genome assembly can be accessed at https://www.ncbi.nlm.nih.gov/nuccore/JAUMTJ000000000.
